# A rare condition that mimic myopathy: Late-onset glutaric acidaemia type II

**DOI:** 10.2478/rir-2023-0026

**Published:** 2023-09-27

**Authors:** Jianwen Liu, Chenmin Wu, Fei Gao, Qing Yan

**Affiliations:** Department of Rheumatology and Immunology, Fujian Provincial Hospital, Shengli Clinical Medical College of Fujian Medical University, Fuzhou, Fujian province, China

Dear Editor,

Inflammatory myositis (IMM) is an autoimmune disease that predomintanely involves proximal limb muscles. Several conditions could “mimic” the clinical pictures of IMM presenting with myalgia, increase of serum muscle enzymes. In this letter,we share a rare case that mimic IMM: Late-onset glutaric acidaemia type II.

A 21-year-old woman visited the emergency department because of muscle weakness and hypoglycaemic coma. She had a 1-year history of exercise intolerance, myalgia and muscle weakness. Her body weight decreased from 65 to 40 kg. Physical examination at admission revealed neck and proximal limb muscle weakness (manual muscle testing 8 [MMT8] score, 26 points) accompanied by severe muscle atrophy. Her serum creatine kinase (CK) and lactate dehydrogenase (LDH) levels were 15514 U/L (reference interval [RI]: 40–200 U/L) and 1438 U/L (RI: 120–250 U/L), respectively, and serum myoglobin (Mb) level was 758.5 ng/mL. Abdominal ultrasonography showed severe hepatic steatosis and electromyography revealed features of myopathy. Magnetic resonance imaging (MRI) of her thigh muscles was normal. The patient was diagnosed with idiopathic inflammatory myositis (IIM), and she was treated with 60 mg/day methylprednisolone (MP) and coenzyme Q10 (10 mg, 3 times/day).

**Figure 1:**
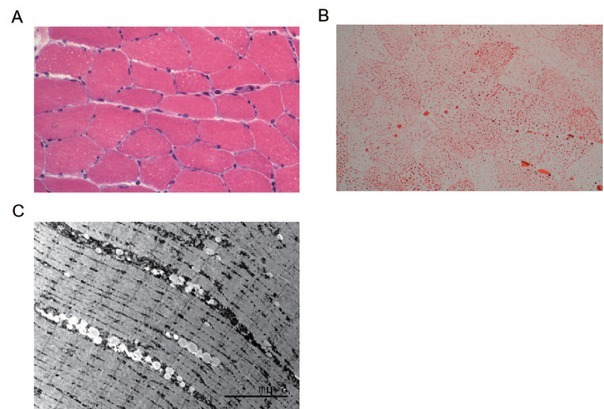
Pathological findings from left quadriceps muscle biopsy in this patient. (A) Varying sizes lipid vacuoles within muscle fibres (HE staining, × 400). (B) Lipid particle deposition within muscle fibres (ORO staining, × 400). (C) Electron microscopy showing lipid droplet deposition within muscle fibres.

Her myalgia and muscle weakness began to improve, and her serum CK level retured to normal range. However, her dysphagia and shortness of breath remained unimproved. Her blood gas test showed higher than normal carbon dioxide partial pressure. Biopsy of the vastus lateralis muscle revealed vacuolar-degenerated myofibres and excess lipid storage (Figure 1A-C). Urine organic acids pyruvate and 3-hydroxybutyrate were detected by Gas chromatographymass spectrometry (GC/MS) which indicated the presence of acetonuria. Inherited metabolic disease tandem mass spectrometry detection was performed and revealed reductions in free carnitine and various acylcarnitines.

**Figure 2:**
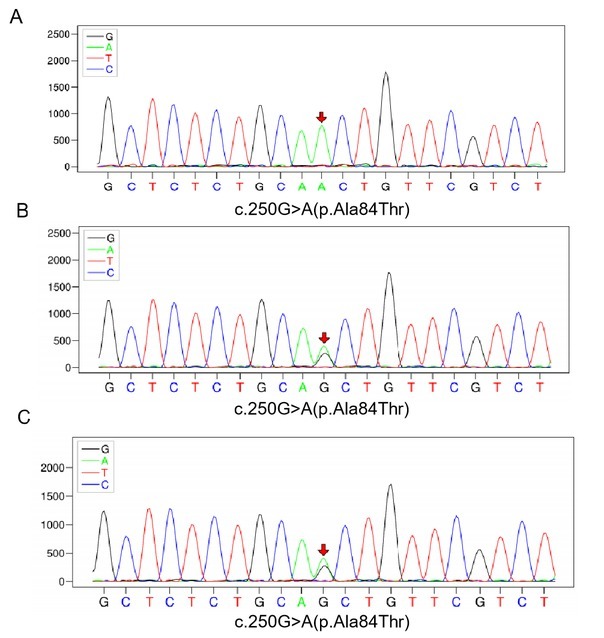
Sequencing of the electron transfer flavoprotein dehydrogenase gene of the patient (A), her father (B), and her mother (C), showed the same missense mutation of c.250G>A (p.Ala84Thr ) in chr4-159603421. The mutation was homozygous, while in her parents it was heterozygous.

Gene analysis was performed thereafter. Homozygous c.250G>A (chr4.159603421) mutation in ETFDH (Figure 2A) was identified. Her parents were both heterozygous for c.250G>A mutation (Figure 2B, C). Thus, the diagnosis of late-onset glutaric acidaemia type II (GAII) was confirmed. Her clinical and biochemical conditions was significantly improved after administration of riboflavin (20 mg three times daily) and L-carnitine (10 mL three times daily).

GAII is an autosomal recessive disorder caused by mutations in genes encoding electron transfer flavoprotein A, B (ETFA, ETFB) or electron transfer flavoprotein dehydrogenase (ETFDH), also known as multiple acyl-coenzyme A dehydrogenase deficiency (MADD), A wide spectrum of different ETFDH mutations has been reported worldwide, of which c.250G > A (p.Ala84Thr) is the most common, being found predominantly in southern China, while c.770A > G (p.Try257Cys) and c.1227A > C (p.Leu409Phe) are more common in northern China. ETFDH mutations often present as late-onset forms. The classical clinical manifestations of late-onset GAII include recurrent or progressive proximal muscle weakness and myalgia accomapined with recurrent episodes of hypoglycaemia with hypoketosis, gastrointestinal dysfunction and hepatic dysfunction. Cold, infection, and nutrient deprivation that increase metabolic stress can exacerbate muscle weakness and rhabdomyolysis.^[1,2]^ However, the symptoms of late-onset GAII are usually atypical, this makes the differential diagnosis difficult. The important hint to differentiate late onset GAII from IMM is that the myositis specific autoantibodies were generally negative in the former condition.^[3]^

The diagnosis of GAII could be made based on the presence of urinary organic acid profiles which is characterized by elevated amounts of glutaric acid, ethylmalonic acid, isovaleric acid, a-methylbutyrate, isobutyrate, aliphatic dicarboxylic acids, and their derivatives, acylcarnitine profiling by tandem mass spectrometry screening of serum or dried blood spot samples characteristically shows increased concentrations of short-, medium-, and long-chain acylcarnitines (C4-C12).^[4]^

In this case, anuria showed by urine analysis and reduced serum acylcarnitine levels were incompatible with the GAII, however, these presentations were consistent with primary systemic carnitine deficiency (CDSP). This may due to fasting before sample collection, malnutrition and residual enzyme activity in the body.^[5]^ Therefore, although acylcarnitine spectrum and gas chromatography-mass spectrometry analysis of organic acids can provide clues for genetic metabolic diseases, the sensitivity and specificity for the diagnosis is low.

Patients with late-onset GAII usually respond to riboflavin, with a response rate as high as 98.4%.^[6]^ However, the dose and course of treatment are controversial, and no systematic evaluation has yet been reported. High-dose and long-term riboflavin treatment is commonly recommended.^[7]^ Furthermore, carnitine supplementation has also been suggested in view of the low carnitine levels in these patients.^[8]^ Glucocorticoids can promote treatment effects to a certain extent in some patients with GAII, but cannot achieve the same efficacy as that of riboflavin.^[9]^

In conclusion, the clinical manifestations of late-onset GAII are generally non-specific and are easily being misdiagnosed especially in the early stage. GAII should be suspected in cases of rapid progression of myopathy, especially in patients with metabolic abnormalities, such as liver damage, hyperammonaemia, and hypoglycaemia. Blood amino acid and acylcarnitine profile analysis, urine organic acid analysis, muscle biopsy, and genetic test should be performed to ensure early diagnosis. Riboflavin supplementation is the first line medication.
